# Highly Efficient Recovery of Ruthenium from Aqueous Solutions by Adsorption Using Dibenzo-30-Crown-10 Doped Chitosan

**DOI:** 10.3390/polym14081551

**Published:** 2022-04-11

**Authors:** Mihaela Ciopec, Oana Grad, Adina Negrea, Narcis Duţeanu, Petru Negrea, Raluca Vodă, Cătălin Ianăşi

**Affiliations:** 1 Faculty of Industrial Chemistry and Environmental Engineering, Polytechnic University of Timişoara, Victoriei Square, No. 2, RO-300006 Timişoara, Romania; mihaela.ciopec@upt.ro (M.C.); oana.grad@upt.ro (O.G.); petru.negrea@upt.ro (P.N.); raluca.voda@upt.ro (R.V.); 2 Institute of Research for Renewable Energies, Strada Musicescu Gavril No. 138, RO-307221 Timișoara, Romania; 3 “Coriolan Drăgulescu” Institute of Chemistry, Bv. Mihai Viteazul, No. 24, RO-300223 Timișoara, Romania; ianasic@acad-icht.tm.edu.ro

**Keywords:** ruthenium, chitosan, dibenzo-30-crown-10, adsorption, desorption, mechanism

## Abstract

Ruthenium, as an industrial by-product or from natural sources, represents an important economical resource due to its specific applications. A complex problem is represented by ruthenium separation during reprocessing operations, therefore, different materials and methods have been proposed. The present study aims to develop a new material with good adsorbent properties able to be used for ruthenium recovery by adsorption from aqueous solutions. Absorbent material was obtained using chitosan (Ch) surface modification with dibenzo-30-crown-10 ether (DB30C10). Chitosan represents a well-known biopolymer with applicability in different adsorptive processes due to the presence of hydroxyl-, carboxyl-, and nitrogen-containing groups in the structure. Additionally, crown ethers are macromolecules with a good complexation capacity for metallic ions. It is expected that the adsorptive efficiency of newly prepared material will be superior to that of the individual components. New synthesized material was characterized using scanning electron microscopy coupled with energy dispersive X-ray (SEM–EDX), Fourier transform infrared spectroscopy (FT-IR), Brunauer–Emmett–Teller surface area analysis (BET), and determination of point of zero charge (pZc). Results obtained from the performed kinetic, thermodynamic, and equilibrium studies confirmed the good adsorptive capacity of the prepared material, Ch-DB30C10, obtaining a maximum adsorption capacity of 52 mg Ru(III) per gram. This adsorption capacity was obtained using a solution with an initial concentration of 275 mg L^−1^, at pH 2, and 298 K. Ru(III) adsorption kinetics were studied by modeling the obtained experimental data with pseudo-first order and pseudo-second order models. Desorption studies established that the optimum eluent was represented by the 5M HNO_3_ solution. Based on the performed studies, a mechanism for recovery of ruthenium by adsorption was proposed.

## 1. Introduction

Extensive usage of nuclear fission in energy production leads to numerous fission atoms with atomic masses between 70 and 160, as components of nuclear radioactive waste. These waste products also contain different valuable elements from the platinum group, such as palladium, rhodium, and ruthenium [1]. Due to its specific properties, ruthenium can be used in different fields: electronic industry, medicine, jewelry, and catalysts, etc. [2,3,4,5]. Other sources of ruthenium can be represented by industrial solutions generated during processing, which contain around 4% metal [6]. Such effluents containing ruthenium ions cannot be discharged as they are harmful to the environment and human health [7].

Different methods for recovering valuable metals from industrial waste is of great interest nowadays. Such methods are: biological treatment [6,8,9], reduction of metallic ions [10], solvent extraction [11,12,13,14], ionic exchange [15], electrochemical deposition [16,17], coprecipitation [18], photo-reduction [11], microfiltration, chemisorption [19,20], etc. Liu et al. [21] demonstrate that several studies were focused on heavy metal elimination by using adsorption on chitosan materials. For example, Metwally et al. [21,22] used chitosan benzoyl thiourea derivatives as adsorbent materials for Co(II) and Eu(III) radionuclide removal from aqueous solutions. Prepared material showed a maximum adsorption capacity of 29.47 mg Co(II) per g and 34.54 mg Eu(III) per g [22]. Cross-linked chitosan was used as adsorbent material for uranium (IV) recovery from aqueous solutions by Wang et al. [21,23]. Santana et al. [21,24] prepared chitosan films using casting techniques and used them further for vanadium recovery obtaining a maximum adsorption capacity of 251.4 mg per g.

Ruthenium recovery by adsorption has a large application due to its efficiency and due to the possibility of processing solutions with relatively low concentrations [25,26,27,28]. In all adsorptive processes, a key factor is represented by the adsorbent material used, being required to develop a proper material. Until now, ruthenium removal by using a chitosan-based adsorbent has been rarely investigated. Liu et al. used xanthate-modified cross-linked chitosan as adsorbent for Ru(III) ions removal [21]. The aim of present study was to produce and characterize a new adsorbent material obtained by functionalization of chitosan (Ch) with dibenzo-30-crown-10 ether (DB30C10). The main reasons for the present study are: Ch represents a bio-polymer with high availability, is eco-friendly, and has active groups (nitrogen-containing groups, hydroxyl and carbonyl groups) able to coordinate metallic ions; crown ethers are a macrocyclic compound able to coordinate metallic cations inside the etheric ring. Due to the presence of the oxygen atoms in the crown ether structure, it is possible that several metallic ions are coordinated.

Based on kinetic, thermodynamic, and equilibrium studies, a mechanism for ruthenium recovery by adsorption on Ch-DB30C10 was proposed. Further desorption studies were performed in order to determine the maximum number of adsorption/desorption cycles.

## 2. Materials and Methods

### 2.1. Material Synthesis and Characterization

For preparation of new adsorbent material, 0.1 g of extractant (DB30C10 with purity of 98%, Merck, Darmstadt, Germany) were weighed, mixed with 25 mL of nitrobenzene (purity 99%, Carl Roth, Karlsruhe, Germany), and shaken until complete dissolution. Extractant solution was placed in contact with 1 g of Ch with a molecular weight between 100,000 and 300,000 (support, ACROS Organics, Geel, Belgium) using a ratio of extractant:support = 0.1 g:1 g. In order to achieve support functionalization, the extractant solution was kept in contact with the support for a minimum of 24 h, filtered, and dried in an oven (Pol-eko model SLW 53, SDT Poland) at 323 K for 24 h.

After preparation, the obtained material was characterized by scanning electron microscopy coupled with energy dispersive X-ray spectroscopy (SEM–EDX) using a scanning electron microscope (FEI Quanta FEG 250, FEI, Hillsboro, Oregon, US) and Fourier-transform infrared spectroscopy (FT-IR) using a Bruker Platinum ATR-QL spectrometer (Bruker, Billerica, Massachusetts, US). The specific material surface was determined using the Brunauer–Emmet–Teller method (BET) with a Nova 1200e Quantachrome device. The point of zero charge (pZc) of the material was determined using the method of bringing the studied system to equilibrium [29,30]. During present study, 0.1 g of Ch-DB30C10 were used, mixed with 25 mL of KCl 0.1 N at 200 rotations per minute and 298 K, using a thermostatic shaker (Julabo SW23, Julabo, Seelbach, Germany). The pH of the KCl solutions was adjusted to the interval 2–12 by using NaOH solutions with concentrations between 0.05 and 2 N, or HNO3 solutions with concentrations between 0.05 and 2 N. After filtration, supernatant pH was measured using a Mettler Toledo SevenCompact S210 pH meter.

### 2.2. Adsorption Studies

During adsorption studies the influence of pH, contact time, temperature, and ruthenium initial concentration on adsorption capacity of Ch-DB30C10 was studied.

#### 2.2.1. pH Influence

pH influence is correlated with the ruthenium oxidation state in the solution and with the functional groups of the extractant. In present paper, the pH influence for the Ru(III) (ruthenium (III) chloride, Fluka Analytical) adsorption on new prepared adsorbent material was studied. During the study, the pH was varied from 0.5 to 10 for an initial concentration of Ru(III) of 10 mg L^−1^, by using 0.1 g of adsorbent material, 120 min of contact time, and 298 K.

#### 2.2.2. Influence of Contact Time and Temperature

In order to establish the influence of contact time and temperature on material adsorption capacity, 0.1 g of prepared adsorbent were weighed and mixed with a 25 mL solution containing 10 mg L^−1^ Ru(III). Each sample was mixed for different times (15, 30, 60, 120, 180, and 240 min), at different temperatures (298, 308, and 318 K), and 200 rpm using a thermostatic bath.

#### 2.2.3. Initial Concentration Influence

To establish how the Ru(III) initial concentration affects the maximum adsorption capacity of Ch-DB30C10, Ru(III) solutions with different concentrations (10, 25, 50, 75, 100, 150, 175, 200, 225, 250 and 275 mg L^−1^) were prepared by using a stock solution with a concentration of 1000 mg L^−1^. In all these cases, the adsorptive process was conducted at pH between 2 and 4 and a temperature of 298 K for 120 min. Ruthenium residual concentration was measured using the ICPOS technique (5100 VDV Agilent Technology, with a double-pass chamber and OneNeb nebulizer). For concentration calculation, the first two most intense spectral lines were used, all concentrations were determined based on 3 replicates, and 5 s integration times.

In order to determine the maximum adsorption capacity of newly prepared adsorbent material, q (mg g^−1^), the following equation was used:

(1) $$q=\frac{\left(C_{0}-C_{f}\right)V}{m}$$

where:

C_0_—initial concentration of Pd (II) from solution, (mg L^−1^);

C_f_—residual concentration of Pd (II) from solution, (mg L^−1^);

V—volume solution, (L);

m—adsorbent mass, (g).

### 2.3. Desorption Studies

The desorption study was conducted in a similar way to the adsorption one. After adsorption of 200 mg L^−1^ Ru(III) on Ch-DB30C10 at pH 2, 298 K, and 200 rpm for 120 min, adsorbent material was dried. Then, 1 g of dried material was placed in contact with 25 mL of different eluents (HNO_3_, HCl, H_2_SO_4_) with different concentrations (0.5, 1, and 5 M) for 60 min at 298 K and 200 rpm.

## 3. Results and Discussion

### 3.1. Material Synthesis and Characterization

After preparation, adsorbent material was characterized by using the physical–chemical methods described earlier.

#### 3.1.1. Scanning Electron Microscopy Coupled with Energy Dispersive X-Ray Spectroscopy (SEM–EDX)

**Figure 1:**
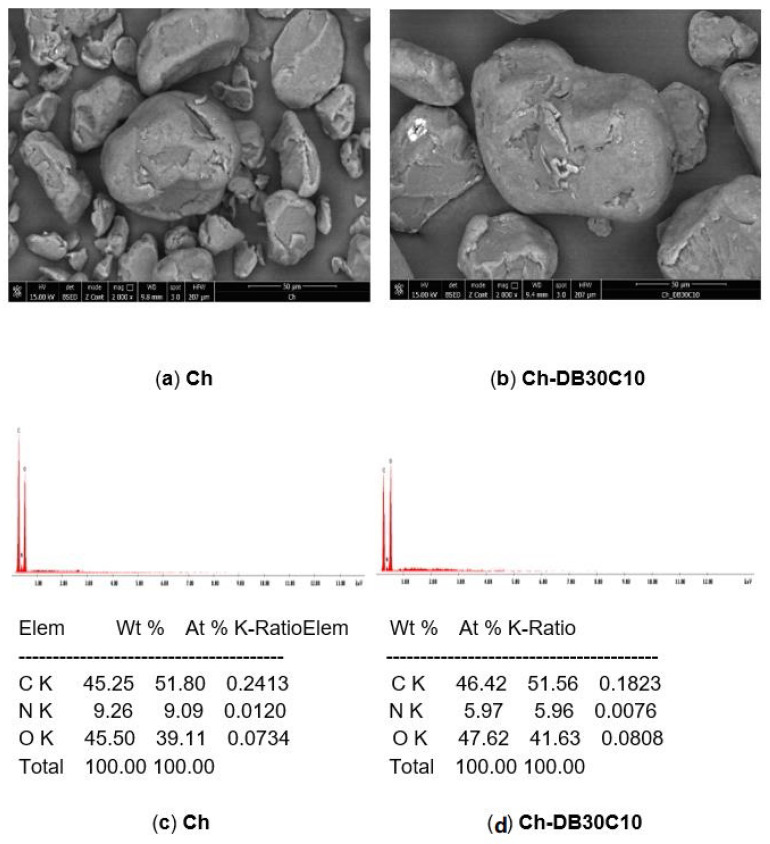
Scanning electron microscopy coupled with energy dispersive X-ray spectroscopy (SEM–EDX). (**a**) SEM recorded for Ch, (**b**) SEM recorded for Ch-DB30C10, (**c**) EDX spectrum recorded for Ch, (**d**) EDX spectrum recorded for Ch-DB30C10.

In Figure 1a,b, the SEM pictures recorded for pure Ch and for Ch-DB30C10 produced adsorbent are depicted and in Figure 1c,d the EDX spectra recorded for pure Ch and for Ch-DB30C10 produced adsorbent are depicted.

Analyzing the image presented in Figure 1a, it can be observed that the support material (Ch) presents a smooth and porous surface. Similar, from the SEM picture depicted in Figure 1b, it can be observed that the prepared adsorbent material (Ch-DB30C10) has a different surface morphology due to the presence of the crown ether (observed as white spots on top of the Ch particles) [31].

The presence of the crown ether on the Ch surface was evidenced also by recording the EDX spectra. In the case of the spectra recorded for produced adsorbent, an increase in the quantities of C and O can be observed, along with the decrease in the quantity of N. This is correlated with the fact that the crown ether has in its structure only C and O atoms.

#### 3.1.2. FT-IR Spectroscopy

**Figure 2:**
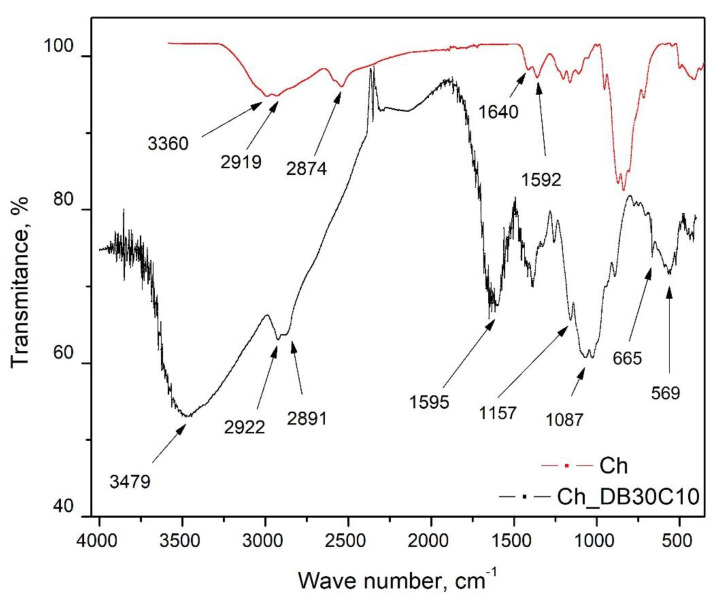
FT-IR spectroscopy.

In Figure 2 the FT-IR spectra recorded for pure Ch and for new prepared adsorbent material (Ch-DB30C10) are presented.

In the spectra recorded for pure Ch, the presence of some characteristic peaks at 3360, 2919, 2874, 1640, 1592, 1375, 1153, 1061, and 893 cm^−1^ can be observed [32]. Vibrations located at 3360 cm^−1^ are specific for –N–H and –O–H bonds. Adsorption bands located at 2919 and 2874 cm^−1^ are attributed to the stretching vibrations of symmetric and asymmetric –C–H bonds, being specific to polysaccharides [33]. The residual N-acetyl group is evidenced by the stretching vibrations observed at 1640 and 1375 cm^−1^. The vibration observed at 1153 cm^−1^ can be attributed to the asymmetric bond –C–O-C–, and the bands located at 1061 and 893 cm-1 correspond to the presence of the -C-O bond [34].

From the FT-IR spectra of Ch-DB30C10, it can be observed that the peaks are located at approximately the same wave numbers, but they are more intense [33]; therefore, confirming Ch functionalization with crown ether.

#### 3.1.3. Determination of the Specific Surface using the BET Method (Brunauer–Emmett–Teller)

**Figure 3:**
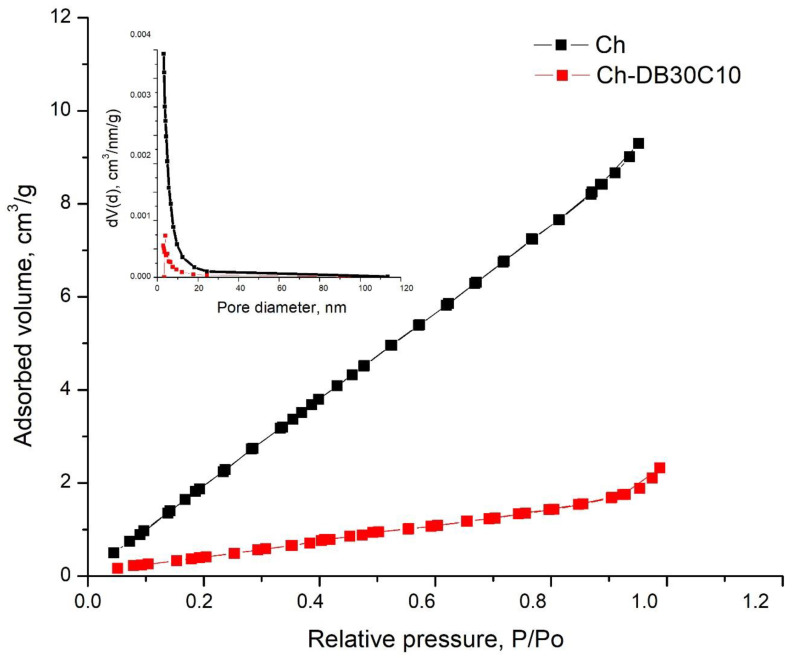
Determination of the specific surface using the BET method.

N_2_ adsorption/desorption isotherms recorded for pure Ch and for Ch-DB30C10 are presented in Figure 3. In the inset graph, the pore size distribution obtained using the BJH method was presented. Both samples indicate a type II isotherm after IUPAC [35].

This type of isotherm is specific for nonporous or macroporous adsorbents.

Analyzing the data obtained from the isotherms, the results indicate an increase in surface area and total pore volume for sample Ch. The textural data obtained for both samples are: raw Ch presents a specific surface area of 15.25 m^2^ g^−1^ and a total pore volume of 0.01493 cm^3^ g^−1^. In the case of newly prepared adsorbent material, a surface area of 1.973 m^2^ g^−1^ and a total pore volume of 0.003599 cm^3^ g^−1^ was obtained.

As a result, from the textural parameters we observed that after the DB30C10 was introduced the pores were filled but the average pore size was not altered. Additionally, it can be stated that pores are filled one by one, so adsorbed material has the tendency to gather sidewards, from where pores are filled. Therefore, no changes were noticed in the material structure, so the pore structure remained unchanged.

#### 3.1.4. Point of Zero Charge, pH_pZc_

**Figure 4:**
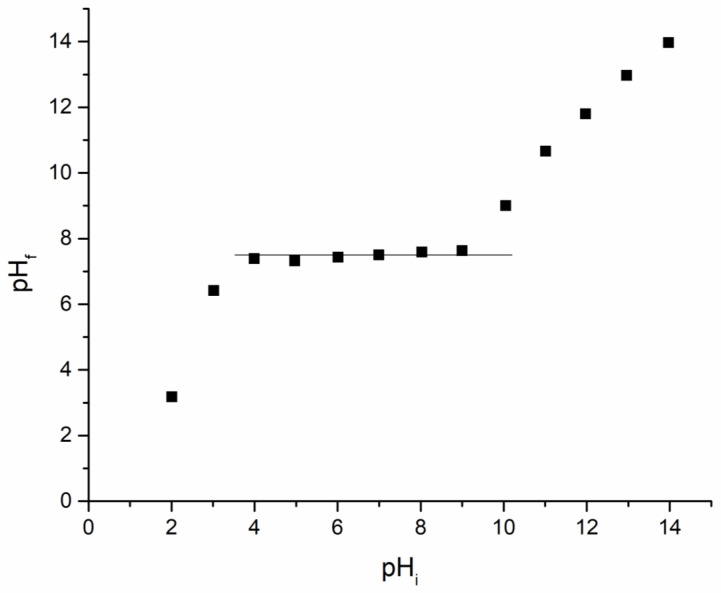
Point of zero charge, pH_pZc_.

One possibility for gathering information about the electrical charge of the Ch-DB30C10 surface is represented by determination of point of zero charge (pH_pzc_). In Figure 4 the graphical representation used for determination of point of zero charge for newly prepared adsorbent material is depicted. Based on experimental data presented in Figure 4, it can be concluded that Ch-DB30C10 point of zero charge is 8. When the solution pH is higher than pH_pzc_, the material surface will be negatively charged, and when pH values are lower it means that the material surface has a positive charge [36,37].

In aqueous solution, at pH lower than 4, the formation of different anionic complexes of Ru(III) was reported, such as [RuCl_4_(H_2_O)_2_]^−^ [38,39], due to the hydrolysis of ruthenium salt. Additionally, it was proved that at a pH higher than 4, ruthenium ions precipitate as Ru(OH)3·nH_2_O, meaning that the Ru(III) recovery must be carried out at a pH lower than 4 [40]. By correlating this observation with the determined pH_pzc_ value, it can be concluded that at pH < 4 it will be adsorbed as an anion complex onto the material surface.

### 3.2. Adsorption Studies

#### 3.2.1. The pH Influence

**Figure 5:**
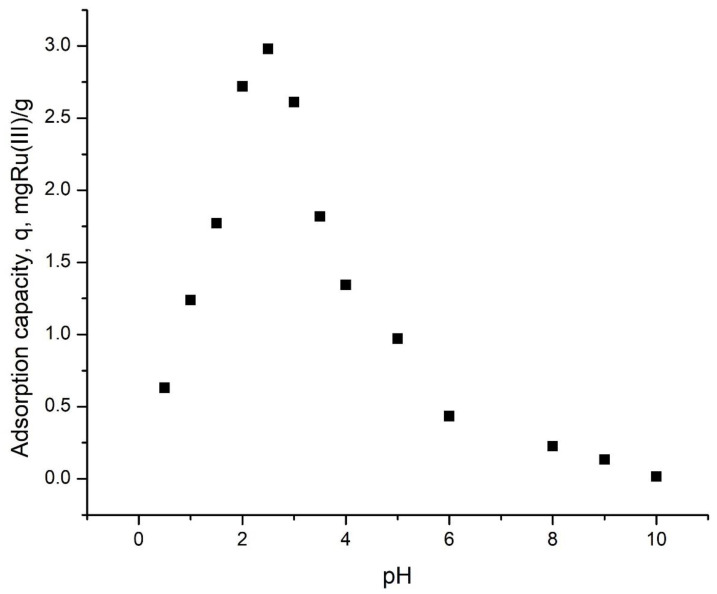
pH influence (data obtained for an initial concentration of 10 mg L^−1^, by using 0.1 g of adsorbent material, 120 min contact time, and 298 K).

One parameter that has a great influence on Ru(III) adsorption is represented by the solution pH, with the obtained data presented in Figure 5.

Analyzing data from Figure 5, it can be observed that the adsorption capacity increases with the increase in the solution pH from 0.5 to 2. At pH 2 the maximum adsorption capacity of around 3 mg Ru(III) per gram of adsorbent material was reached. Further increase in the pH leads to a decrease in the adsorption capacity. This behavior must be correlated with the complex behavior of Ru ions in aqueous solutions, where those ions suffer from hydrolysis with formation of different complexes. Marenich et al. [41] proved that at acidic pH, ruthenium forms aqua-complexes, such as Ru^III^(H_2_O)_18_^3+^. At this point, by correlating the data from Figure 5 with the information regarding the RuCl_3_ hydrolysis, it can be concluded that the recovery of such ions from aqueous solutions must be carried out at pH 2.

#### 3.2.2. Influence of the Contact Time and Temperature

**Figure 6:**
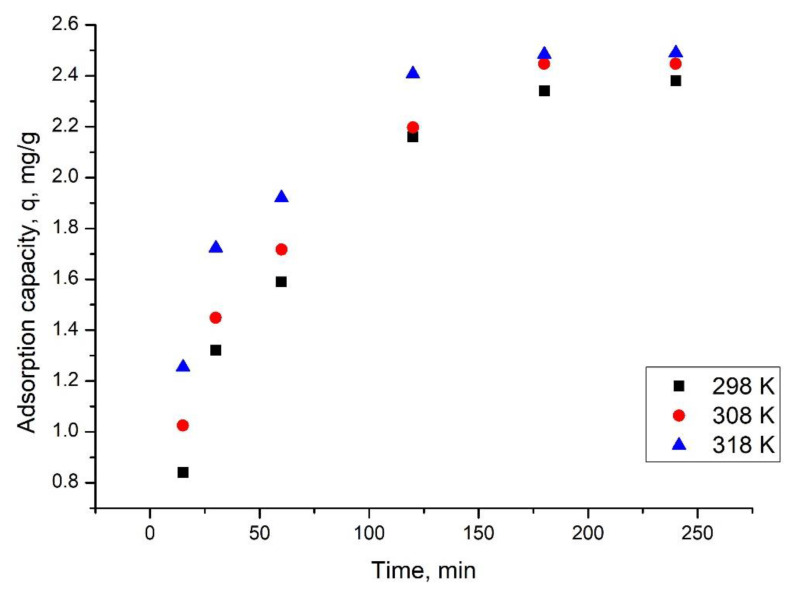
Contact time and temperature influence (0.1 g of adsorbent, 25 mL solution containing 10 mg L^−1^ Ru(III), pH 2).

Other important parameters for adsorptive processes are represented by the contact time and working temperature. In the present paper, the influence of these two parameters on the Ru(III) adsorption on Ch-DB30C10 was followed. In Figure 6, the dependence between maximum adsorption capacity and contact time at three different temperatures is depicted.

From data presented in Figure 6, it can be observed that at all temperature values the increase in the contact time until 120 min has a beneficial effect, leading to an increase in the maximum adsorption capacity. For any further increase in the contact time, the adsorption capacity remains relatively constant at approximatively 2.5 mg Ru(III) adsorbed per each gram of adsorbent material. Based on these observations, it can be concluded that the optimum contact time is 120 min. It can also be observed that the increase in the temperature leads to no significant increase in the maximum adsorption capacity (from 2.5 mg g^−1^ at 298 K, to 2.6 mg g^−1^ at 318 K), so any further experiments were carried out at 298 K.

#### 3.2.3. The Ru(III) Initial Concentration Influence

**Figure 7:**
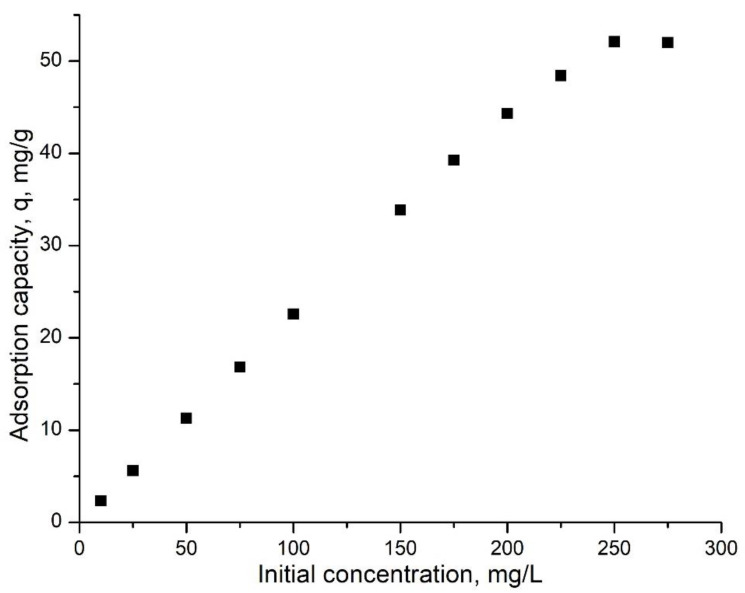
The Ru(III) initial concentration influence (pH 2, contact time 120 min, 298 K).

Likewise, the influence of the Ru(III) initial concentration on its adsorption process on Ch-DB30C10 was studied. In Figure 7 the dependence between the maximum adsorption capacity and Ru(III) initial concentration is presented.

From the data presented in Figure 7, an increase in maximum adsorption capacity as well as the increase in Ru(III) initial concentration can be observed until 250 mg per liter. Further increase in the initial concentration leads to no increase in the maximum adsorption capacity. Therefore, it can be concluded that the maximum adsorption capacity of approximately 52 mg Ru(III) per gram of adsorbent material was obtained for an initial concentration of 250 mg Ru(III) per liter. Experimental data proved that the raw Ch exhibited a maximum adsorption capacity of 17.5 mg Ru(III) per g, respectively, and crown ether presented a maximum adsorption capacity of 36.3 mg Ru(III) per g. In this context, it can clearly be observed that the prepared material is superior to the individual components.

#### 3.2.4. Kinetic Studies

In order to determine the kinetics of studied process, the Lagergren model (pseudo-first order model) and Ho and McKay model (pseudo-second order model) were used. The kinetic equation used to describe the pseudo-first order model is [42]:

$$\ln\left(q_{e}-q_{t}\right)={\mathrm{lnq}}_{e}-k_{1}t$$

where: 

q_e_—adsorption capacity at equilibrium (mg/g);

q_t_—adsorption capacity at t time (mg/g);

k_1_—pseudo-order-one speed constant (1/min);

t—contact time (min).

A similar mathematical equation used to describe the pseudo-second order model is [43,44,45]:

$$\frac{t}{q_{t}}=\frac{1}{k_{2}q_{e}^{2}}+\frac{t}{q_{e}}$$

where: 

q_e_—adsorption capacity at equilibrium (mg/g);

q_t_—adsorption capacity at t time (mg/g);

k_2_—pseudo-order-two speed constant (g/mg∙min);

t—contact time (min).

**Figure 8:**
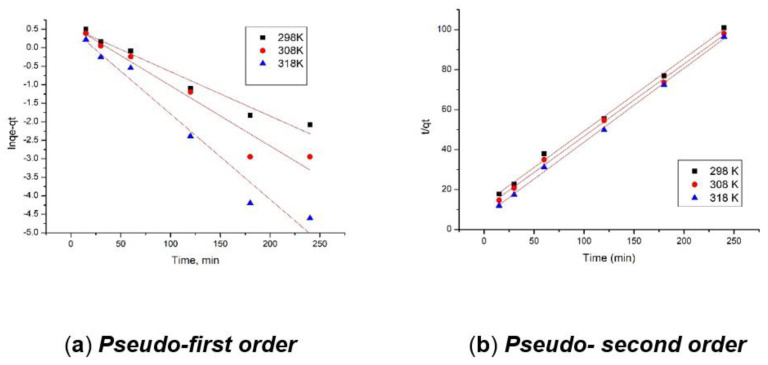
Pseudo-first order (**a**) and pseudo-second order (**b**) kinetic isotherms.

Parameters associated with the kinetic models used were determined from graphical representations of linearized forms of mathematical equations associated with the models. In the case of the pseudo-first order model, the linear dependence between ln(q_e_ − q_t_) and t was represented and from the line equation the values of k_1_ and q_e calc_ were determined. Additionally, when the experimental data were modeled using the pseudo-second order model, the linear dependence between t/qt and t was represented, and from the line equation the values for parameters k_2_ and q_e calc_ were determined. In the present paper, the kinetics of Ru(III) adsorption on Ch-DB30C10 was studied at three different temperatures: 298, 308, and 318 K. The obtained pseudo-first order and pseudo-secondorder kinetic isotherms are presented in Figure 8. Based on the depicted kinetic isotherms, the speed constants, maximum adsorption capacities, and regression coefficients were calculated, which are presented in Table 1.

Analyzing the data presented in Figure 8 and Table 1, it can be observed that the experimental data are modeled with higher accuracy by the pseudo-second order model. This fact is supported by the R^2^ (0.9965–0.9979) values closer to unity, and by the better alignment of points with the linear form of the pseudo-second order model. By comparison, when the obtained experimental data were modeled using the pseudo-first order model, values of R^2^ were located between 0.9716 and 0.9887. Likewise, the values of maximum adsorption capacities using these kinetic models were estimated. In the case of the pseudo-second order model, at the temperature of 298 K a value of approximately 2.43 mg Ru(IIII) per gram of adsorbent was obtained, which is closer to the experimental value of 2.34 mg Ru(III) per gram of adsorbent. Experimental data proved that the temperature has no significant influence over the maximum adsorption capacity, so in this case, there is no need to work at a higher temperature. In order to understand whether film diffusion or intraparticle diffusion represent the speed determining step, the obtained experimental data were modeled using the Weber–Morris model. This model is described by the following mathematical equation:

q_t_ = k_diff_ • t^1/2^ + C

where: 

q_t_—adsorption capacity at time t;

k_diff_—intraparticle diffusion speed constant, mg/g·min^1/2^;

C—constant correlated with the thickness of the liquid film surrounding the adsorbent particles.

**Figure 9:**
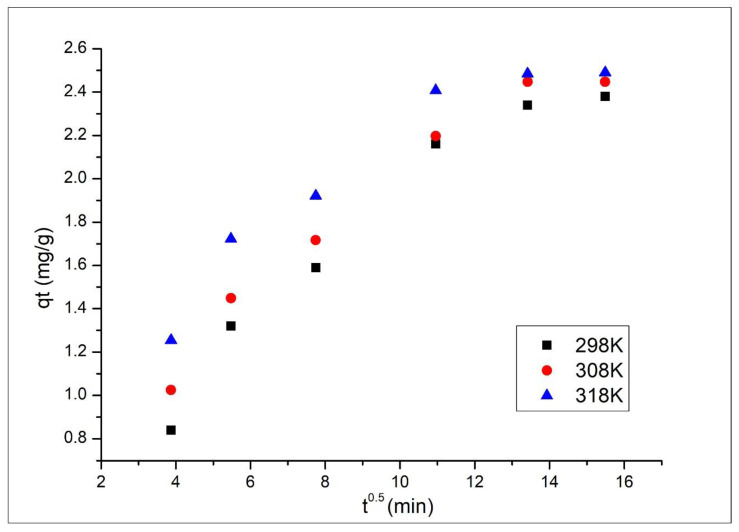
The intraparticle diffusion model parameters for the adsorption of Ru onto Ch-DB30C10 material at different temperatures.

Curves obtained after modeling the experimental data with the Weber–Morris model are presented in Figure 9.

From the data depicted in Figure 9, it can be observed that the curves representing the dependence q_t_ versus t^1/2^ do not pass through the origin. Such behavior is associated with a multi-step adsorptive process, which means that both the film and intraparticle diffusion can influence the adsorption kinetics. Numerical values obtained for parameters k_diff_ and C associated with the Weber–Morris model are presented in Table 2.

From the data presented in Table 2, it can be observed that the value of k_diff_ increases with the increase in the working temperature. Additionally, it can be observed that the diffusion constants for the first stage are larger than the constants associated with the second stage. Based on that, it can be concluded that the first stage is determines the global adsorption speed and the second one limits the overall process [2].

Further, for Ru(III) adsorption on Ch-DB30C10, the activation energy was calculated using the Arrhenius equation and the speed constant was obtained using the pseudo-second order model.

$$\ln k_{2}=\mathrm{lnA}-\frac{E_{a}}{\mathrm{RT}}$$

where:

k_2_—speed constant (g/min∙mg);

A—Arrhenius constant (g∙min/mg);

E_a_—activation energy (kJ/mol);

T—absolute temperature (K);

R—the ideal gas constant (8.314 J/mol∙K).

**Figure 10:**
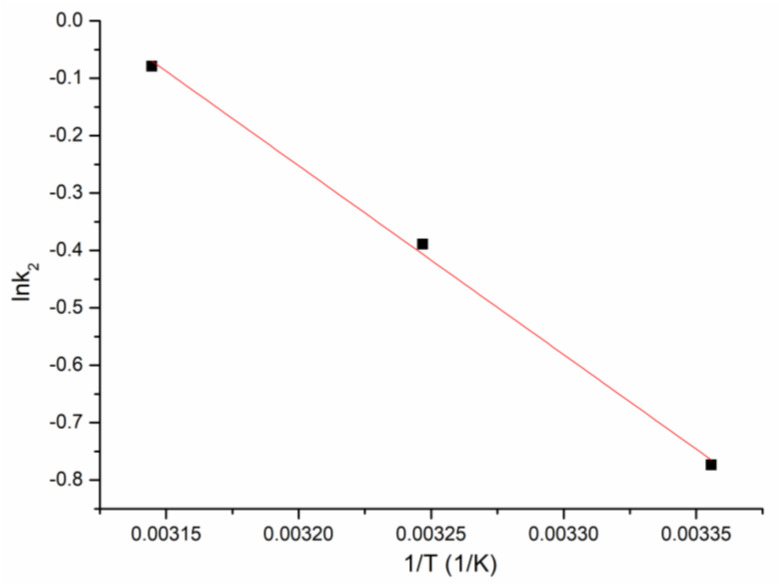
The ln k_2_ vs. 1/T plot.

The value of the activation energy indicates whether the studied adsorptive process is physical or chemical. In the case of Ru(III) adsorption on Ch-DB30C10, the value of the activation energy was determined from the linear dependence between lnk_2_ and 1/T (depicted in Figure 10). For this process, the activation energy has a value of 27.3 kJ mol^−1^, meaning that the Ru(III) adsorption is a physical adsorption (value lower than 40 kJ mol^−1^) [46].

#### 3.2.5. Thermodynamic Studies

**Figure 11:**
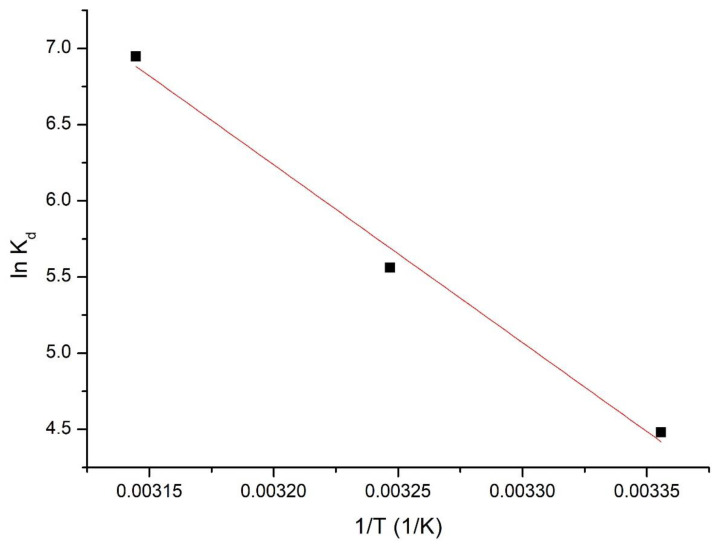
The ln Kd vs. 1/T plot.

Thermodynamic studies were performed in order to determine the following thermodynamic parameters: Δ*G°*, Δ*H°*, and Δ*S°*. All of these studies were performed in the temperature range between 298 and 318 K. Firstly, based on the van’t Hoff equation and from the linear dependence between ln K_d_ versus 1/T (depicted in Figure 11), the values of enthalpy standard variation (Δ*H°*) and entropy standard variation (Δ*S°*) were determined. Further, the value of free Gibbs energy was determined using the Gibbs–Helmholtz equation [47].

Obtained values of the thermodynamic parameters are presented in Table 3.

Analyzing the information presented in Table 3, it can be observed that the value of Δ*H°* is positive, meaning that the studied adsorption process is endodermic. For the free Gibbs energy, negative values were observed, which increased with the increase in the temperature, meaning that the process is spontaneous and influenced by temperature. Positive values of Δ*S°* indicate that the adsorptive process is favorable and is taking place at the interface of the Ch-DB30C10/Ru(III) solution.

#### 3.2.6. Equilibrium Studies

In the next step, the mechanism of the adsorption process was established by modeling the obtained experimental data with three specific isotherms: Langmuir, Freundlich, and Sips.

The Langmuir isotherm [48] was used to model the obtained experimental data, in order to evaluate the maximum adsorption capacity of the prepared adsorbent material. The nonlinear expression of the Langmuir isotherm is:

$$q_{e}=\frac{q_{L}K_{L}C_{e}}{1+K_{L}C_{e}},$$

where:

*q_L_*—Langmuir maximum adsorption capacity (mg/g);

*K_L_*—Langmuir constant.

The Freundlich isotherm assumes that the surface of the adsorbent material used is heterogeneous, so it can be further considered that the distribution of the adsorption heat on such surface is uneven and multilayer adsorption can occur due to the presence of an unlimited number of active centers.

The Freundlich isotherm is an empirical isotherm [49] given by the relationship:

$$q_{e}=K_{F}C_{e}^{1/n_{F}}$$

where *K_F_* and *n_F_*—the characteristic constants that can be related to the relative adsorption capacity of the adsorbent and the intensity of adsorption.

The Sips isotherm [50] was derived from the Langmuir and Freundlich isotherms. In the case of low adsorbate concentrations, it reduces to the Freundlich isotherm, and if the adsorbate concentrations are high, it exhibits the characteristics of the Langmuir isotherm. Therefore, this isotherm can be used to compute the adsorption capacity. The nonlinear equation of the Sips isotherm is:

$$q_{e}=\frac{q_{S}K_{S}C_{e}^{1/n_{S}}}{1+K_{s}C_{e}^{1/n_{S}}}\;,$$

where:

*K_S_*—constant related to the adsorption capacity of the adsorbent;

*n_S_*—the heterogeneity factor.

**Figure 12:**
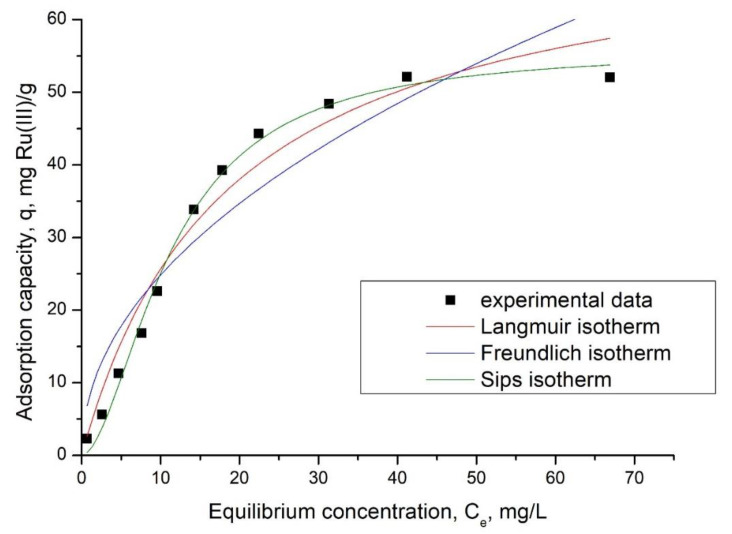
Adsorption isotherms.

In Figure 12, the obtained adsorption isotherms are presented.

From the slopes of the straight lines of the adsorption isotherms represented in Figure 12, specific parameters were determined for each isotherm used for modeling the experimental data (Table 4).

From the data presented in Table 4, it can be observed that Sips isotherm accurately describes Ru(III) adsorption on Ch-DB30C10, due to the regression coefficient being closer to 1 [2], and due to the value of adsorption capacity being closer to the experimental one. Because the studied adsorption is described by the Sips isotherm, it can be concluded that this can take place as a multi-layer adsorption.

In Table 5, the adsorption capacities obtained for the adsorption of Ru(III) on different adsorbent materials are presented. Based on these data, it can be concluded that Ch-DB30C10 has the best performance for Ru(III) adsorption.

### 3.3. Desorption Behavior

An important aspect is represented by the possibility of reusing the adsorbent material. Therefore, in this context the desorption of adsorbed Ru using different eluents, such as HNO_3_, HCl, and H_2_SO_4_, was studied with concentrations between 0.1 and 5 M. Obtained experimental data are depicted in Table 6. From the obtained experimental data, it can be observed that the increase in the eluent concentration leads at increase in desorption efficiency. Additionally, it can be observed that the maximum desorption efficiency of 96.7 was obtained when HNO_3_ 5 M was used as the eluent.

### 3.4. Proposal of a Mechanism for the Ru(III) Adsorption Process

**Figure 13:**
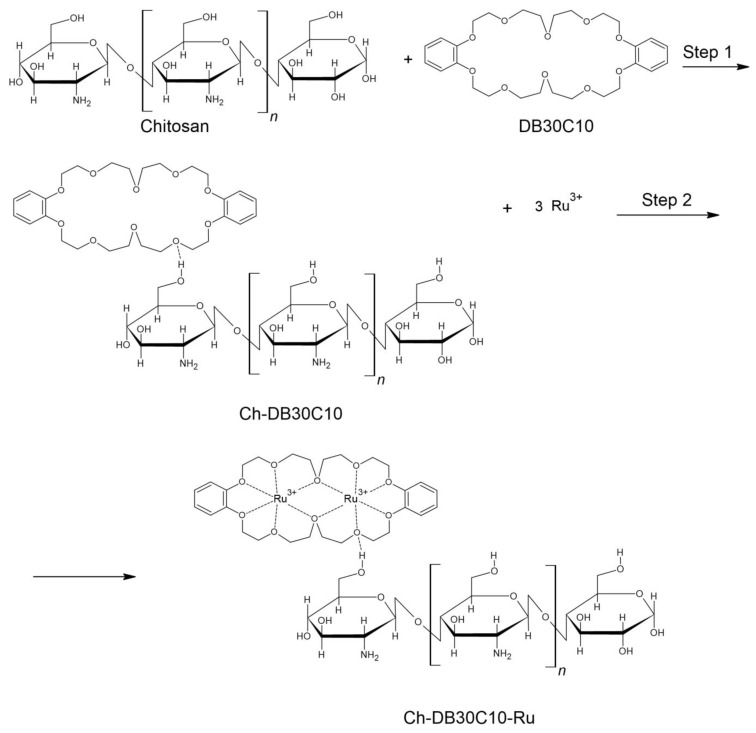
Mechanism for Ru(III) adsorption onto Ch-DB30C10.

Based on the performed studies, the following mechanism for Ru(III) adsorption onto Ch-DB30C10 (Figure 13) was proposed.

## 4. Conclusions

In present study, a new adsorbent material was prepared using chitosan functionalization with dibenzo-30-crown-10 ether, and the obtained material had good efficiency for Ru(III) adsorption for aqueous solutions. The best results were obtained when the process was undertaken at pH 2, contact time of 120 min, and 298 K. By using the optimum conditions, kinetic, thermodynamic, and equilibrium studies were performed.

From the kinetic studies, it was determined that the pseudo-second order model was the best out of the studied processes. In order to make a clear distinction between film and intra-particle diffusion, experimental data were modeled using the Weber–Morris model. The porous structure of Ch-DB30C10 active sites can also be located inside the channels, but experimental data indicated that the Ru(III) ions are adsorbed inside the crown ether. In this case, it can be concluded that the metallic ions are adsorbed as film and the intra-particle diffusion is not a limiting factor for the studied adsorption process. Based on the value of activation energy, it was established that Ru(III) adsorption is a physical process. Based on the obtained experimental data, it was observed that the maximum adsorption capacity of Ch-DB30C10 was 52 mg Ru(III) per gram of adsorbent material.

In order to establish whether the prepared material can be reused, desorption tests were performed using different eluents. From these, 5 M HNO_3_ represents the optimum one for Ru(III) recovery (96.7%). After desorption, the adsorbent material was used for a new recovery cycle. The maximum number of adsorption/desorption cycles until the adsorbent material was exhausted was 11. Based on the obtained data, an adsorption mechanism was proposed.

It can be concluded that the newly prepared adsorbent material can be used for further recovery of Ru(III) ions from aqueous solutions by adsorption.

## Figures and Tables

**Table 1:** Kinetic parameters for the adsorption of Ru(III) onto Ch-DB30C10.

Pseudo-First Order				
Temperature (K)	*q*_e,exp_ (mg/g)	*k*_1_ (1/min)	*q*_e,calc_ (mg/g)	R^2^
298	2.34	0.012	1.73	0.9808
308	2.44	0.013	1.61	0.9887
318	2.50	0.023	1.68	0.9716
**Pseudo-second order**				
**Temperature (K)**	***q*_e,exp_** **(mg/g)**	***k*_2_** **(g/mg∙min)**	***q*_e,calc_** **(mg/g)**	**R^2^**
298	2.34	0.461	2.43	0.9971
308	2.44	0.677	2.49	0.9979
318	2.50	0.923	2.60	0.9965

**Table 2:** The intraparticle diffusion model parameters for the adsorption of Ru(III) onto Ch-DB30C10.

Intraparticle Diffusion Model						
Temperature (K)	K_1diff_ (mg/g·min^1/2^)	C_1_	R^2^	K_2diff_ (mg/g·min^1/2^)	C_2_	R^2^
298	0.402	0.108	0.9237	0.174	0.0186	0.8979
308	1.611	0.139	0.9231	0.383	0.0492	0.7989
318	2.216	0.166	0.8912	0.585	0.0566	0.8986

**Table 3:** Thermodynamic parameters for adsorption of Ru(III) onto Ch-DB30C10.

Δ*H*º (kJ/mol)	Δ*S*º (J/mol∙K)	Δ*G*º (kJ/mol)			R^2^
		298 K	308 K	318 K	
97.06	362.4	−10.9	−14.56	−18.19	0.9981

**Table 4:** Parameters of the isotherm model for adsorption of Ru(III) onto Ch-DB30C10.

Langmuir Isotherm			
*q*_m,exp_ (mg/g)	*K*_L_ (L/mg)	*q*_L_ (mg/g)	*R* ^2^
52.1	0.053	73.6	0.9626
Freundlich isotherm			
*K*_F_ (mg/g)	1/*n*_F_		*R* ^2^
8.19	0,481		0.8764
Sips isotherm			
*K*_S_	*q*_S_ (mg/g)	1/*n*_S_	*R* ^2^
0.013	55.9	0.78	0.9931

**Table 5:** Comparison of adsorption performance with other materials for Ru(III).

Adsorbent Material	pH	T (K)	C_0_ (mg L^−1^)	q_m_ (mg g^−1^)	Reference
Raw C. glutamicum biomass	2.5–2.7	293	61.6	16	[9]
Lewatit MonoPlus M600	2.5–2.7	293	61.6	6.7	[9]
Ion-imprinted blend membrane (Ru(III)-IIM)	2	298	60	44.1	[51]
PNSBs	2	338	3250	40	[52]
Amberjet 4200	2.5–2.7	298	1000	31.2	[26]
NIM	2	298	60	20.6	[51]
M500	2.5–2.7	298	3250	17.9	[26]
TRPO/SiO_2_-P	1	298	1059.8	54.6	[2]
Ch-DB30C10	2	298	275	52	This work

**Table 6:** Desorption efficiency of Ru(III) using various eluents.

Eluent Concentration	HNO_3_			HCl			H_2_SO_4_		
	0.5 M	1 M	5 M	0.5 M	1 M	5 M	0.5 M	1 M	5 M
Efficiency, %	85.6	92.4	96.7	78.7	81.2	87.4	45.6	76.8	79.8

## Data Availability

Not applicable.

## References

[1] Bush R.P. (1991). Recovery of platinum group metals from high level radioactive waste. Platinum Met. Rev..

[2] Zhang S., Ning S., Liu H., Zhou J., Wang S., Zhang W., Wang X., Wei Y. (2020). Highly-efficient separation and recovery of ruthenium from electroplating wastewater by a mesoporous silica-polymer based adsorbent. Microporous Mesoporous Mater..

[3] Das N. (2010). Recovery of precious metals through biosorption—A review. Hydrometallurgy.

[4] Yan Y., Wang Q., Xiang Z., Yang Y. (2018). Separation of Pt(IV), Pd(II), Ru(III), and Rh(III) from chloride medium using liquid–liquid extraction with mixed imidazolium-based ionic liquids. Sep. Sci. Technol..

[5] Won S.W., Kwak I.S., Mao J., Yun Y.-S. (2015). Biosorption–Incineration–Leaching–Smelting Sequential Process for Ru Recovery from Ru-Bearing Acetic Acid Waste Solution. Ind. Eng. Chem. Res..

[6] Noah N.F.M., Othman N., Jusoh N. (2016). Highly selective transport of palladium from electroplating wastewater using emulsion liquid membrane process. J. Taiwan Inst. Chem. Eng..

[7] Zhang H., Yang X., Liu Z., Yang Y. (2017). Recovery of Ru(III) from hydrochloric acid by cloud point extraction with 2-Mercaptobenzothiazole-functionalized ionic liquid. Chem. Eng. J..

[8] Dobson R.S., Burgess J.E. (2007). Biological treatment of precious metal refinery wastewater: A review. Miner. Eng..

[9] Kwak I.S., Won S.W., Chung Y.S., Yun Y.-S. (2013). Ruthenium recovery from acetic acid waste water through sorption with bacterial biosorbent fibers. Bioresour. Technol..

[10] Aktas S., Morcali M.H., Aksu K., Aksoy B. (2017). Recovery of Ruthenium Via Zinc in the Presence of Accelerator. Trans. Indian Inst. Met..

[11] Li F., Shang Y., Ding Z., Weng H., Xiao J., Lin M. (2017). Efficient extraction and separation of palladium (Pd) and ruthenium (Ru) from simulated HLLW by photoreduction. Sep. Purif. Technol..

[12] Moeyaert P., Miguirditchian M., Masson M., Dinh B., Hérès X., De Sio S., Sorel C. (2017). Experimental and modelling study of ruthenium extraction with tri-n-butylphosphate in the purex process. Chem. Eng. Sci..

[13] Rzelewska M., Wiśniewski M., Regel-Rosocka M. (2017). Effect of composition and ageing of chloride solutions on extraction of Rh(III) and Ru(III) with phosphonium ionic liquids Cyphos IL 101 and IL 104. Sep. Sci. Technol..

[14] Tateno H., Park K.C., Tsukahara T. (2018). Direct Extraction of Platinum Group Metals from Nitric Acid Solution Using the Phase Transition of Poly(N-isopropylacrylamide). Chem. Lett..

[15] Kurimura Y., Nagashima M., Takato K., Tsuchida E., Kaneko M., Yamada A. (1982). Photoredox reactions using ion-exchange resin-adsorbed Ru(bpy)32+. Photosensitized reductions of methyl viologen and molecular oxygen using ion-exchange resin-adsorbed tris(2,2′-bipyridine)ruthenium(II). J. Phys. Chem..

[16] Song Y., Tsuchida Y., Matsumiya M., Tsunashima K. (2018). Recovery of ruthenium by solvent extraction and direct electrodeposition using ionic liquid solution. Hydrometallurgy.

[17] Vereecken P.M., Radisic A., Ross F.M. (2018). Differential Inhibition during Cu Electrodeposition on Ru: Combined Electrochemical and Real-Time TEM Studies. J. Electrochem. Soc..

[18] Minamisawa H., Kuroki H., Arai N., Okutani T. (1999). Coprecipitation of ruthenium with chitosan and its determination by graphite furnace atomic absorption spectrometry. Anal. Chim. Acta.

[19] Zeng J., Zhang Z., Zhou H., Liu G., Liu Y., Zeng L., Jian J., Yuan Z. (2019). Ion-imprinted poly(methyl methacrylate-vinyl pyrrolidone)/poly(vinylidene fluoride) blending membranes for selective removal of ruthenium(III) from acidic water solutions. Polym. Adv. Technol..

[20] Ito T., Nagaishi R., Kimura T., Kim S.-Y. (2015). Study on radiation effects on (MOTDGA–TOA)/SiO_2_-P adsorbent for separation of platinum group metals from high-level radioactive waste. J. Radioanal. Nucl. Chem..

[21] Liu S., Li Y., Yan J., Zhou Y., Liu C., Zuo Y., Liu D. (2021). Effective removal of ruthenium(III) ions from wastewater by xanthate-modified cross-linked chitosan. J. Environ. Chem. Eng..

[22] Metwally E., Elkholy S., Salem H., Elsabee M. (2009). Sorption behavior of 60Co and 152+154Eu radionuclides onto chitosan derivatives. Carbohydr. Polym..

[23] Wang G., Liu J., Wang X., Xie Z., Deng N. (2009). Adsorption of uranium (VI) from aqueous solution onto cross-linked chitosan. J. Hazard. Mater..

[24] Santana Cadaval T.R., Camara A.S., Dotto G.L., Pinto L.A.d.A. (2013). Adsorption of Cr (VI) by chitosan with different deacetylation degrees. Desalin. Water Treat..

[25] Godlewska-Żyłkiewicz B., Zambrzycka-Szelewa E., Leśniewska B., Wilczewska A. (2012). Separation of ruthenium from environmental samples on polymeric sorbent based on imprinted Ru(III)-allyl acetoacetate complex. Talanta.

[26] Kim S., Choi Y.-E., Yun Y.-S. (2016). Ruthenium recovery from acetic acid industrial effluent using chemically stable and high-performance polyethylenimine-coated polysulfone-Escherichia coli biomass composite fibers. J. Hazard. Mater..

[27] Ramesh A., Hasegawa H., Sugimoto W., Maki T., Ueda K. (2008). Adsorption of gold(III), platinum(IV) and palladium(II) onto glycine modified crosslinked chitosan resin. Bioresour. Technol..

[28] Zhang A., Xu L., Lei G. (2016). Separation and complexation of palladium(ii) with a new soft N-donor ligand 6,6′-bis(5,6-dinonyl-1,2,4-triazin-3-yl)-2,2′-bipyridine (C9-BTBP) in nitric acid medium. New J. Chem..

[29] Borah D., Satokawa S., Kato S., Kojima T. (2009). Sorption of As(V) from aqueous solution using acid modified carbon black. J. Hazard. Mater..

[30] Borah D., Satokawa S., Kato S., Kojima T. (2008). Surface-modified carbon black for As(V) removal. J. Colloid Interface Sci..

[31] Kumar S., Koh J. (2012). Physiochemical, Optical and Biological Activity of Chitosan-Chromone Derivative for Biomedical Applications. Int. J. Mol. Sci..

[32] Kumar S., Koh J., Kim H., Gupta M.K., Dutta P.K. (2012). A new chitosan–thymine conjugate: Synthesis, characterization and biological activity. Int. J. Biol. Macromol..

[33] Grad O., Ciopec M., Negrea A., Duțeanu N., Vlase G., Negrea P., Dumitrescu C., Vlase T., Vodă R. (2021). Precious metals recovery from aqueous solutions using a new adsorbent material. Sci. Rep..

[34] Vino A.B., Ramasamy P., Shanmugam V., Shanmugam A. (2012). Extraction, characterization and in vitro antioxidative potential of chitosan and sulfated chitosan from Cuttlebone of *Sepia aculeata* Orbigny, 1848. Asian Pac. J. Trop. Biomed..

[35] Thommes M., Kaneko K., Neimark A.V., Olivier J.P., Rodriguez-Reinoso F., Rouquerol J., Sing K.S.W. (2015). Physisorption of gases, with special reference to the evaluation of surface area and pore size distribution (IUPAC Technical Report). Pure Appl. Chem..

[36] Zhang L., Hu P., Wang J., Huang R. (2016). Crosslinked quaternized chitosan/bentonite composite for the removal of Amino black 10B from aqueous solutions. Int. J. Biol. Macromol..

[37] Kausar A., Naeem K., Hussain T., Nazli Z.-I., Bhatti H.N., Jubeen F., Nazir A., Iqbal M. (2018). Preparation and characterization of chitosan/clay composite for direct Rose FRN dye removal from aqueous media: Comparison of linear and non-linear regression methods. J. Mater. Res. Technol..

[38] Balcerzak M. (2002). Analytical Methods for the Determination of Ruthenium: The State of the Art. Crit. Rev. Anal. Chem..

[39] Rard J.A. (1985). Chemistry and thermodynamics of ruthenium and some of its inorganic compounds and aqueous species. Chem. Rev..

[40] Musić S., Ristic M. (1987). Adsorption of microamounts of ruthenium on hydrous iron oxides. J. Radioanal. Nucl. Chem. Artic..

[41] Marenich A.V., Majumdar A., Lenz M., Cramer C.J., Truhlar D.G. (2012). Construction of Pourbaix Diagrams for Ruthenium-Based Water-Oxidation Catalysts by Density Functional Theory. Angew. Chem. Int. Ed..

[42] Lagergren S. (1898). About the theory of so-called adsorption of soluble substabces. Kungl. Sven. Vetenskapsakad. Handlingarl.

[43] Ho Y.-S. (2006). Review of second-order models for adsorption systems. J. Hazard. Mater..

[44] Ho Y.S., McKay G. (1998). A Comparison of Chemisorption Kinetic Models Applied to Pollutant Removal on Various Sorbents. Process. Saf. Environ. Prot..

[45] Ho Y.S., Mckay G. (1998). The kinetics of sorption of basic dyes from aqueous solution by sphagnum moss peat. Can. J. Chem. Eng..

[46] Zhang Y., Yu F., Cheng W., Wang J., Ma J. (2017). Adsorption Equilibrium and Kinetics of the Removal of Ammoniacal Nitrogen by Zeolite X/Activated Carbon Composite Synthesized from Elutrilithe. J. Chem..

[47] Atkins P., de Paula J. (2005). Atkins’ Physical Chemistry.

[48] Langmuir I. (1918). The adsorption of gases on plane surfaces of glass, mica and platinum. J. Am. Chem. Soc..

[49] Freundlich H.M.F. (1906). Over the adsorption in solution. J. Phys. Chem..

[50] Sips R. (1948). On the Structure of a Catalyst Surface. J. Chem. Phys..

[51] Zeng J., Zhang Z., Dong Z., Ren P., Li Y., Liu X. (2017). Fabrication and characterization of an ion-imprinted membrane via blending poly(methyl methacrylate-co-2-hydroxyethyl methacrylate) with polyvinylidene fluoride for selective adsorption of Ru(III). React. Funct. Polym..

[52] Colica G., Caparrotta S., De Philippis R. (2012). Selective biosorption and recovery of Ruthenium from industrial effluents with *Rhodopseudomonas palustris* strains. Appl. Microbiol. Biotechnol..

